# PROTOCOL: Outreach programmes to improve life circumstances and prevent further adverse developmental trajectories of at‐risk youth in OECD countries: A systematic review

**DOI:** 10.1002/cl2.1121

**Published:** 2020-12-15

**Authors:** Trine Filges, Nina T. Dalgaard, Bjørn C. A. Viinholt

**Affiliations:** ^1^ VIVE—The Danish Center for Social Science Research Copenhagen Denmark

## 1 BACKGROUND

### 1.1 The problem, condition or issue

At‐risk youth may be defined as a diverse group of young people in unstable life circumstances, who are currently experiencing or are at risk of developing one or more serious problems such as school failure or drop‐out, mental health disorders, substance and/or alcohol abuse, unemployment, long‐term poverty, delinquency and more serious criminal behaviour (Arbreton et al., 2005; Quinn, 1999). At‐risk youth typically have a multitude of social and psychological problems and typically also come from families considered at‐risk (Treskon, 2016). They may occasionally or permanently be homeless and spend time in the streets.

No readily available statistics on the numbers of at‐risk youth exist but statistics on the numbers experiencing the adverse outcomes can be found. For example, according to the National Conference of State Legislatures (NCSL) on any given night, approximately 41,000 unaccompanied youth ages 13–25 experience homelessness in the United States (NCSL, 2019). It is estimated that 4.2 million youth and young adults experience homelessness each year, and that 10% of young adults ages 18–25, and at least one in 30 adolescents ages 13–17, experience some form of homelessness over the course of a year (NCSL, 2019). A substantial part of them report having a number of other problems too; for example, having substance misuse problems (29%), mental health problems (69%) or been in the juvenile justice system, in jail or detention (50%), Further, school drop‐out and no high school diploma or General Equivalency Diploma is the number one correlate for elevated risk of youth homelessness (NCSL, 2019). In Denmark the numbers are much lower. The estimated number of homeless youth, <25 years of age, was 1,036 in 2019 (Benjaminsen, 2019) which amounts to <1% of those aged 13–24 years; but in line with the evidence from the United States a large part of them have other problems (e.g., substance misuse and mental health problems) as well and the majority in the age group 18–24 are NEET, that is, neither employed nor in education or training (Benjaminsen et al., 2020). Numbers of homeless youth across Organisation for Economic Co‐operation and Development (OECD) countries are hard to locate and definitions of homelessness vary across countries (OECD, 2020a) but most likely, there is as great variation as in other indicators of at‐risk youth. For example, the rates of school drop‐out, those that do not reach a basic minimum level of skills, is on average 19% across OECD countries and range from 2% in Korea to 58% in Turkey for the 25–34 years old (OECD, 2012). Also, the NEET rates vary a lot across OECD countries; from <7% of the 15–29 year old in Iceland and the Netherlands to more than 37% in South Africa with an OECD average of 13% (OECD, 2020b).

At‐risk youth are often very unlikely to seek out help for themselves within the established venues, as their adverse developmental trajectories have installed a lack of thrust in authorities such as child protection agencies and social workers (Ronel, 2006). In order to help this population, a number of outreach programmes have been established seeking to help the young people on an ad hoc basis, meaning that the interventions are designed to fit the individual needs of each young person rather than as a one‐size‐fits‐all treatment model (Korf et al., 1999; Svensson et al., 2003). The programmes are often multicomponent interventions and often rely on volunteers as outreach workers, as these are proposed to offer the young people a unique possibility for forming trusting relationships due to the fact that help is offered as an act of altruism (Ronel, 2006). The programmes may offer basic necessities such as food or shelter and they may offer counselling, mentoring and medical assistance. What define the outreach programmes is that they are targeted at helping the young people away from the streets and their current adverse developmental paths towards more stable living situations and developmental prospects.

Due to the very nature of the programmes, the effects are difficult to determine. First, randomisation is difficult when there is no system of referral, and the uniquely tailored interventions, which each young person receives raises the question if one can even describe the intervention as uniform even within the same programme. Second, the aims of the programmes are typically to change the long‐term developmental paths of the participants, but longitudinal studies are often not feasible, and the establishment of long‐term preventive effects is difficult. However, even if the obstacles are many, it is still important to explore the efficacy of outreach programmes, as the stakes are extremely high. If left alone, the target population of at‐risk youth are likely to develop serious long‐term problems, which are not just detrimental to the individual but also very costly to societies.

### 1.2 The intervention

The intervention in this review is targeted outreach work which may be (but does not have to be) multicomponent programmes in which outreach may be combined with other services. There are different meanings of the concept outreach work throughout Europe and a wide variety of outreach initiatives with different arrangements were outreach may work in one or many ways (Svensson, 2003).

The term *outreach work* as we will use it in this review is commonly known throughout Scandinavia and is corresponding with detached youth work in England (similar to street work or fieldwork; Korf et al., 1999). Detached outreach work is executed outside any agency setting, is taking place in the community where groups of marginalised youth are known to meet, with the aim of engaging young people who lack any kind of belonging by directing young people to treatment or care services when necessary. It may be based on voluntary efforts, peer groups or professionals, social workers, social pedagogical workers and health workers but the common nature is to meet the young people on their own terms. Outreach work is based on voluntary participation and is an important approach for intervening with hard to reach populations, and identifying their needs in a flexible and responsive manner with no manual based restrictions.

However, an outreach programme may be associated with a specific service or combination of services offered by one or more organisations targeting a specific population. The services combined with the outreach component could be case management or participation in community programmes or even a continuum of comprehensive services including education, employment, and intensive supervision.

Outreach efforts with services only focusing on nutritional and medical care (e.g., testing for human immunodeficiency virus [HIV]) will be excluded.

The comparison population are young people at‐risk who are not contacted by the outreach workers and are not encouraged to attend any services.

### 1.3 How the intervention might work

The primary mechanism of change in outreach work with at‐risk youth is to facilitate positive change by gradually building up a sense of trust between the young person and the outreach worker(s) (Svensson, 2003). Characteristically, the aim of the outreach youth worker is to find solutions to young people's problems in their own environment, rather than deciding while sitting behind a desk what they consider best for the person concerned. The goal is always to prevent further marginalisation and encourage social integration (Svensson, 2003).

Theoretically, outreach work may be understood through an empowerment lens. Empowerment theory is both a value orientation for working in the community and a theoretical model for understanding the processes whereby individuals gain access to resources and acquire skills and knowledge enabling them to take advantage of opportunities within the community and to exert control and influence over decisions that affect their lives (Zimmerman, 2002). As a value orientation empowerment theory proposes that many social problems exist because of unequal distribution of, and access to, resources within the community. The theory further suggests that many individuals are best served by mutual help, helping others or working for their rights rather than having their needs fulfilled by a benevolent professional (Perkins & Zimmerman, 1995; Zimmerman, 2002). What this means is that outreach work is aimed at enabling the at‐risk young person to function more autonomously and adaptively within their community rather than just providing a quick fix for their current problems. Empowerment theory proposes that by identifying strengths rather than pointing out and cataloguing risk factors, at‐risk youth may become motivated to actively engage in their own positive change. Outreach work may thus also be understood as aimed at promoting resiliency by enabling the young person to make better use of their personal and social resources. Theoretically a number of protective factors may serve to buffer the adversity a young person might be exposed to. Protective factors at the personal level may include being physically healthy, having a good self‐esteem and adaptive coping skills. At the family level protective factors may include having a supportive network of family or friends and at the societal level protective factors may include living in a community with acces to support. Thus, outreach work may be seen as drawing on resiliency theory when working to assist the young person in identifying protective factors (Zimmerman et al., 2013). As proposed by Rappaport (1985) social change based on empowerment is proposed to be brought on by a change of both language and conceptions. Instead of perceiving the outreach workers and at‐risk young people as “professionals” and “clients”, empowerment thinking proposes a bidirectional relationship between helpers and participants. In outreach work this means that the outreach workers aim to meet the at‐risk youth with a none‐judgemental approach characterised by genuine empathy rather than prejudice and victim blaming (Svensson, 2003; Zimmerman, 2002). In addition to meeting the youth with empathy outreach workers strive to become “culturally competent” which may be defined as the willingness to understand young people from different cultural and social backgrounds and the ability to put oneself in their situation. It also includes the ability and readiness to sympathise with young people subjected to prejudice, social exclusion and stigmatisation, and to approach each young person with respect, open‐mindedness and commitment (Svensson, 2003).

As stated in the introduction at‐risk youth often come from socioeconomically less advantaged and dysfunctional families (Treskon, 2016). At risk youth have often experienced at number of adverse events such as poverty, emotional or physical abuse and neglect, out‐of‐home placement, living with mentally ill or substance abusing parents and unstable housing situations leading to a lack of continuity in their education. Thus, at‐risk youth often lack stable attachment figures and suitable adult role models, which leads to a lack of adaptive life skills and compromises their ability to seek appropriate help within established venues. Early adverse experiences may also lead to a deeply installed mistrust of authorities and thus at‐risk youth are often unlikely to seek out help for themselves. In line with empowerment thinking, outreach programmes seek to meet the young person at their own terms offering them the specific help they need here and now and thus slowly building up a trusting relationship which may be used for future motivational work (Svensson, 2003). Outreach workers aim at offering the young person a positive adult role model and thus provide the young person with the kind of socioemotional support which they often lack. Sometimes outreach workers may teach the young person basic life skills, such as personal hygiene, offer assistance with home work or writing job applications, paying bills, getting help for substance or alcohol abuse problems and being on time for work or school, or they may accompany the young person to meetings with authority figures, which are fear‐inducing in the young person due to their negative past experiences. Furthermore, outreach work may include tutoring programmes, or offer assistance with baby‐sitting and housing for socially disadvantaged teenage mothers. What characterises all efforts is that they seek to support and install a sense of empowerment within the young person which may enable them to master similar challenges in the future in a more adaptive way and to motivate the young person to behaviour changes which may facilitate further social reintegration (Perkins & Zimmerman, 1995; Svensson, 2003; Zimmerman, 2002).

In sum, empowerment theory provides a framework for understanding the mechanisms of change within youth outreach work. The goal of outreach work with at‐risk youth is to facilitate positive long‐term social change by motivating the young person to become actively engaged. Based on Svensson (2003) the theoretical approach to youth outreach work is based on the following principles:

– Distribution of services where youth, subcultural groups, young people at risk and young drug users are present in their own environment.
– To design services based on the needs young people demonstrate and encourage their voluntary participation.
– The outreach work is based on voluntary relations between the youth and the outreach worker. The relation is based on confidence, distinctness and continuity.
– The outreach work is executed on the young people's own terms.
– Respect for the youth's own values, their needs, their civil and human rights, their choice and their responsibility for their own lives. Meet people with nonjudgemental attitude, integrity, frankness and honesty.

### 1.4 Why it is important to do this review

We have located one systematic review on outreach programmes for youth; however, it only included programmes for street‐involved youth, a term used by the authors instead of homeless youth (Connolly & Joly, 2012). The participant population was young people aged 12–25, who did not have a permanent place of residence. Furthermore, it only included articles published in peer‐review journals between 1990 and 2010 and had no restrictions on how the studies measured an impact (i.e., studies without comparison groups were included). The only impact result reported is on later participation rates in the offered service.

Further, we have located five systematic reviews on street‐connected and/or homeless youth.

The systematic review by Coren et al. (2016), focused on street‐connected children and young people (i.e., living on, or closely connected to, the street), from birth to 24 years, and included studies of harm reduction or reintegration interventions that used a comparison group study design. The searches were performed up to April 2015. The primary outcomes of the review were inclusion and reintegration. The secondary outcomes were measures of health, well‐being and educational and occupational achievement. Thirteen studies were included and most of them compared therapy‐based services versus usual shelter and drop‐in services, or versus other therapeutic/health interventions.

Another systematic review on homeless youth (between the ages of 12–24 years) focused solely on HIV/acquired immunodeficiency syndrome prevention programmes (Naranbhai et al., 2011). The searches were performed up to December 2010 and only randomised controlled trials were included.

In the systematic review by Altena et al. (2010), studies published up to 2008 were included if they empirically examined the effectiveness of an intervention for homeless youth. Randomised as well as nonrandomised studies and studies without a control group, that is, before‐after studies were included. No meta‐analysis was performed, only a narrative analysis describing each study and results.

The systematic review by Slesnick et al. (2009), included runaway, shelter, street or drop‐in centre recruited youth between the ages of 12–24. In addition to intervention studies, the review also included studies assessing youth outcomes after shelter or drop‐in utilisation (i.e., service evaluations) and qualitative studies. No meta‐analysis was performed, only a narrative analysis describing each study and results. When the searches were performed is not reported.

In Xiang (2013), studies that examined the effectiveness of interventions to improve substance abuse problems among homeless youth between the ages of 12 and 24 were included. Searches were performed up to April 2012. Only studies that reported data on substance use outcomes were included. Randomised as well as nonrandomised studies and studies without a control group, that is, before‐after studies were included. No meta‐analysis was performed, only a narrative analysis describing each study and results.

Three systematic reviews were found, focusing explicitly on mentoring interventions for youth.

Tolan et al. (2008) performed a systematic review on mentoring intervention with the aim of affecting juvenile delinquency and associated problems for youth, defined as persons under age 18. The review was limited to studies conducted within the United States or another predominately English‐speaking country reported between 1970 and 2005. Eligible outcomes were measures of juvenile delinquency, aggression or high levels of externalising problems, drug abuse and academic achievement/school failure.

DuBois et al. (2002) searched for studies from 1970 through 1998 reporting on the effectiveness of one‐on‐one mentoring programmes for youth. The eligible age of youth is not reported but the average age of the youth participants in the study population had to be <19. The review included before‐after studies, and excluded studies were the adult mentors were mental health professionals (e.g., social workers). Studies of peer tutoring or mentoring programmes were also excluded. It is unclear what the eligible outcomes were, all outcomes was analysed in one meta‐analysis; however a moderator analysis distinguishes between the outcome types: emotional/psychological, problem/high‐risk behaviour, social competence, academic/educational and career/employment.

DuBois et al. (2011) is a follow up to DuBois et al. (2002) with some modifications. Before‐after studies were no longer eligible, participants was required to be <18 years, studies of peer tutoring or mentoring programmes were now eligible and mentoring was not required to be one‐on‐one. The review included studies published between 1999 and 2010. Eligible outcomes were attitudinal/motivational, social/relational, psychological/emotional, conduct problems, academic/school, physical health and career/employment.

Besides being up to date, a major difference between these nine systematic reviews and the current proposal is, that we will focus on programmes with a targeted outreach component for youth aged 8–25.Participants need not be homeless (but are eligible if they are), and we will only include studies with a control group. All relevant outcome areas will be analysed separately in a meta‐analysis taking into consideration the dependencies between effect sizes.

#### 1.4.1 Policy relevance

Public as well as private after‐school programmes and youth clubs that provide healthy alternatives for youth have been shown to serve as important resources for reducing school failure and youth crime (Parker, 2011). However, it is questionable whether the youth who would benefit most are those who are attracted to and attend such programmes (Arbreton & McClanahan, 2002). Outreach work represents an important preventive working approach with the aim of attracting and serving the youth who are very unlikely to participate on their own and who probably need help the most.

Outreach programmes targeting at‐risk youth are designed to reach the youth who need help to prevent high‐school dropout, crime, drug abuse, and other forms of delinquency. Besides the nonmonetary costs in terms of pain, suffering, and lost quality of life the youth incur themselves, there are potentially large financial costs to society that can be saved. A 1998 study estimated the total costs to society of allowing one youth to leave high school for a life of crime and drug abuse to be somewhere between $1.7 and $2.3 million (Cohen, 1998). There are thus more than one good reason to put more weight on prevention efforts.

## 2 OBJECTIVES

The main objective of this review is to answer the following research questions: What are the effects of outreach programmes on problem/high‐risk behaviour of young people between 8 and 25 years of age living in OECD countries? Are they less likely to experience an adverse outcome such as school failure or drop‐out, runaway and homelessness, substance and/or alcohol abuse, unemployment, long‐term poverty, delinquency and more serious criminal behaviour?

## 3 METHODS

### 3.1 Criteria for considering studies for this review

#### 3.1.1 Types of studies

The proposed project will follow standard procedures for conducting systematic reviews using meta‐analysis techniques.

Due to ethical considerations, it is hard to imagine that a researcher would control the allocation of youth at risk to outreach and no outreach. We therefore anticipate that relatively few controlled trials on the effects of outreach on the problem/risk behaviour of at‐risk youth will be found. However, in the unlikely event that a controlled trial is found, it will of course be included in the review.

In order to summarise what is known about the possible causal effects of outreach, we will include all study designs that use a well‐defined control group. Nonrandomised studies, where outreach has occurred in the course of usual decisions outside the researcher's control, must demonstrate pretreatment group equivalence via matching, statistical controls or evidence of equivalence on key risk variables and participant characteristics. These factors will be outlined in the protocol, and the methodological appropriateness of the included studies will be assessed according to a risk of bias model.

The study designs we will include in the review are:

1. Controlled trials (where all parts of the study are prospective, such as identification of participants, assessment of baseline, and allocation to intervention, and which may be randomised or nonrandomised), assessment of outcomes and generation of hypotheses (Higgins & Green, 2011).
2. Nonrandomised studies (outreach has occurred in the course of usual decisions, the allocation to outreach and no outreach is not controlled by the researcher, and there is a comparison of two or more groups of participants, that is, at least a treated group and a control group).

Nonrandomised studies using an instrumental variable approach will not be included—see the Appendix (*Justification of exclusion of studies using an instrumental variable (IV) approach*) for our rationale for excluding studies of these designs.

#### 3.1.2 Types of participants

The review will include young people between 8 and 25 years of age living in OECD countries, who either have experienced or is at‐risk of experiencing an adverse outcome such as school failure or drop‐out, runaway and homelessness, substance and/or alcohol abuse, unemployment, long‐term poverty, delinquency/criminal behaviour.

At‐risk may be based on such indicators as the young person's level of association with negative peers (e.g., negative attitudes toward school and poor educational outlook, gang members, etc.), hanging out on the streets or in gang neighbourhoods, poor academic history, coming from a highly distressed or crisis ridden, low income family in a racially/ethnically segregated neighbourhood, and prior involvement in illegal and delinquent activities.

Studies where the majority of participants are between 8 and 25 years of age will be included.

#### 3.1.3 Types of interventions

The intervention in this review are targeted outreach work which may be combined with other services. There are different meanings of the concept outreach work throughout Europe (Svensson, 2003). The term *outreach work* as we will use it in this review is commonly known throughout Scandinavia and is corresponding with detached youth work in England (similar to street work or fieldwork, Korf et al., 1999). Detached outreach work is executed outside any agency setting, is taking place in the community where groups of marginalised youth are known to meet, with the aim of engaging young people who lack any kind of belonging, and directing young people to treatment or care services when necessary. An outreach programme may be associated with a specific service or combination of services offered by one or more organisations targeting a specific population. The services combined with the outreach component could be case management or participation in community programmes or even a continuum of comprehensive services including education, employment and intensive supervision.

Outreach efforts with services only focusing on nutritional and medical care (e.g., testing for HIV) will be excluded.

The comparison population are young people at‐risk who are not contacted and encouraged by the outreach workers to attend any services.

#### 3.1.4 Types of outcome measures

The primary outcome is problem/high‐risk behaviour, as the overall review question is to evaluate current evidence on outreach programmes' effects on problem/high‐risk behaviour for young people who have experienced or are at risk of experiencing an adverse outcome. We seek evidence on how to best reduce or eliminate problem/high‐risk behaviour, as problem/high‐risk behaviour is understood as the young people's primary problem.

All measures will be included, that is, we do not require that measures have been standardised on a different population.

##### Primary outcomes

The primary focus is on measures of problem/high‐risk behaviour, such as delinquency/criminal behaviour, drug and alcohol use, high levels of externalising problems, school failure, sexual risk taking, gang involvement/membership, poverty, unemployment, runaway and homelessness.

##### Secondary outcomes

A secondary focus is on measures of social and emotional outcomes, such as internalising symptoms (anxiety, depression), self‐identity, interpersonal relations and social awareness

###### Adverse outcomes

Any adverse effects of interventions will be included as an outcome including a worsening of outcome on any of the included measures. Other adverse effects could be, for example, measured by rates of hospitalisation, suicide and over‐doses.

####### Duration of follow‐up

We will include outcomes measured during and after intervention as well as follow‐up at any given point in time.

####### Types of settings

Detached outreach work is executed outside any agency setting, is carried out in the community where groups of marginalised youth are known to meet, with the aim of engaging young people who lack any kind of belonging, and attracting young people to treatment or care services when necessary.

Distribution of outreach services thus takes place where youth, subcultural groups, young people at risk and young drug users are present in their own environment.

Furthermore, we will include outreach services delivered in any format meaning that we will include services that are delivered at an individual level (that includes conversation, adult contacts, following up and being available), at a group level (the outreach worker relates to different youth groups and gangs, and initiates in‐group activities) and finally local community work (such as finding places for the young people to spend their spare‐time, contact and collaboration with other youth workers and between voluntary and public organisations when that is suitable).

### 3.2 Search methods for identification of studies

Relevant studies will be identified through searches in electronic databases, governmental and grey literature repositories, hand search in specific targeted journals, citation tracking, contact to international experts and internet search engines.

#### 3.2.1 Electronic searches

The following electronic databases will be searched:

- ERIC (EBSCO)
- Academic Search (EBSCO)
- EconLit (EBSCO)
- PsycINFO (EBSCO)
- International Bibliography of the Social Sciences (ProQuest)
- Sociological Abstracts (ProQuest)
- Science Citation Index Expanded (Web Of Science)
- Social Sciences Citation Index (Web Of Science)

##### Description of the search‐string

The search string is based on the PICO(s)‐model, and contains two concepts, of which we have developed two corresponding search facets: population characteristics and the intervention. The search string includes searches in title, abstract and subject terms for each facet. The subject terms in the facets will be chosen according to the options available in each database.

Below is an exemplified search string from the database SocIndex. The search string is structured in the following order:

- Search 1–4 covers the intervention
- Search 5–8 covers the population characteristics
- Search 9 combines the two search facets

Interface—EBSCOhost Research Databases. Database—SocINDEX with Full Text.

**Table:** 

Search	Terms	Results
S9	S4 AND S8	855
S8	S5 OR S6 OR S7	119,970
S7	AB (“at‐risk” OR “school dropout*” OR “school failure*” OR “mental disorder*” OR abus* OR poverty OR crim* OR homeless* OR street* OR detached* OR delinquen*) AND AB (child* OR youth OR adolescent* OR young OR teen* OR student*)	99,921
S6	TI (at‐risk OR detached*)	28,092
S5	DE “AT‐risk youth”	334
S4	S1 OR S2 OR S3	6,287
S3	AB (outreach* OR “street work” OR “fieldwork” OR “youth work”) AND AB (program* OR service* OR mentor* OR “social worker*” OR initiative* OR project*)	4,761
S2	TI (outreach* OR “street work” OR “fieldwork” OR “youth work”)	1,914
S1	DE “OUTREACH programs”	372

##### Limitations of the search‐string

No limitations will be implemented in the database searches.

#### 3.2.2 Searching other resources

##### Hand‐search

We will conduct a hand search of specific journals, in order to make sure that all relevant articles are found. The hand search will focus on editions published between 2018 and 2020 in order to secure recently unpublished articles which have not yet been indexed in the bibliographic databases. We will decide upon which journals to hand search based on the identified records from the electronic searches. The following are examples of specific journals which we may decide to hand search:

- Children and Youth Services
- The Future of Children
- Research on Social Work Practice
- Journal of Prevention and Intervention in the Community

##### Searches for unpublished literature in general

Most of the resources searched for unpublished literature include multiple types of references. As an example, the resources listed to identify reports from national bibliographical resources also include working papers and dissertations, as well as peer‐reviewed references. In general, there is a great amount of overlap between the types of references in the chosen resources. The resources are listed once under the category of literature we expect to be most prevalent in the resource, even though multiple types of unpublished/published literature might be identified in the resource.

Further resources for identifying dissertations might be added during the search process. A final list of resources will be included in the appendix of the review.

##### Search for dissertations

We will search the following resources for dissertations:

- ProQuest Dissertations and Theses Global (ProQuest)
- EBSCO Open Dissertations (EBSCO‐host)

##### Search for working papers/conference proceedings

We will search the following resources for working papers/conference proceedings:

- American Institutes for Research (AIR): https://www.air.org/
- Manpower Demonstration Research Corporation (MDRC): https://www.mdrc.org/
- Urban Institute: https://www.urban.org/

##### Search for reports

Public/Private Ventures (P/PV) publications (although P/PV has ceased operations, its publications is archived with the Foundation Center's IssueLab: https://ppv.issuelab.org

##### Search for non‐US literature

We will search the following resources for non‐US literature:

- Danish National Research Database: http://www.forskningsdatabasen.dk/en
- SwePub—Academic publications at Swedish universities: http://swepub.kb.se/
- NORA—Norwegian Open Research Archives: http://nora.openaccess.no/
- CORE—research outputs from international repositories: https://core.ac.uk/
- Skolporten—Swedish Dissertations: https://www.skolporten.se/forskning/
- DIVA–Digital Scientific Archives: http://www.diva-portal.org/smash/

##### Search for systematic reviews

If we identify relevant systematic reviews during the search process, they will be used for citation‐tracking, in order to extract relevant references from the review.

##### Citation‐tracking

We will use citation‐tracking methods to identify more relevant literature. We will citation‐track forwards (by using Google Scholar and Web of Science) and backwards (by screening citations in the most relevant literature).

##### Contact to experts

We will contact international experts to identify unpublished and ongoing studies

### 3.3 Data collection and analysis

#### 3.3.1 Description of methods used in primary research

Randomised controlled trials are eligible but we expect that almost all studies will be conducted without randomisation of participants. Studies of the effect of outreach work are required to have a control group for inclusion in the review. Participants may be allocated by, for example, time differences, location differences, decision makers, policy rules or participant preferences. They must all demonstrate pretreatment group equivalence via matching, statistical controls, or evidence of equivalence on key risk variables and participant characteristics. The methodological appropriateness will be assessed according to the risk of bias model outlined in Section 4.3.5. The risk of bias assessment makes it possible to discriminate between studies with varying degrees of risk. Studies that have been coded with a critical risk of bias will not be included in the data synthesis.

An example of a study that may be included is McClanahan et al. (2012) who evaluated the Philadelphia‐based Youth Violence Reduction Partnership (YVRP) programme. The YVRP employs proactive strategies aimed at addressing the root causes of violence by providing high‐risk youth with intensive supervision and positive support among other services using street workers. YVRP targets youth who are on active probation, typically between the ages of 14 and 24 and live in one of the six YVRP police districts. McClanahan et al. (2012) compared programme youth to youth who resided outside the geographical programme boundaries and were similar to programme youth on background characteristics (e.g., age, history of involvement in the juvenile justice or criminal justice system, family history of abuse and neglect, sibling involvement in the juvenile justice or criminal justice system). Propensity scores were estimated and used to create the comparison group by matching individuals on the estimate of their likelihood of participating in the programme.

Another example is Campie et al. (2014) who evaluated the Massachusetts Safe and Successful Youth Initiative (SSYI), an initiative launched in 11 cities with the highest per capita rates of violent crime. SSYI aims to reduce violence and promote healthy development and outcomes among young males, ages 14–24 who are at the greatest risk for violent offending and victimisation. Campie et al. (2014) estimated propensity scores to measure an individual's probability of selection and participation in the programme (only males aged 14–24 were eligible and they further included variables for whether or not the young male had committed a gun crime or had committed a knife crime). Besides matching individuals on the estimate of their likelihood of participating in the programme they further statistically controlled for age, race, gun crime perpetration and knife crime perpetration.

#### 3.3.2 Criteria for determination of independent findings

In order to determine the independence of results in included studies, we will consider whether individuals may have undergone multiple interventions, whether there were multiple treatment groups and whether several studies are based on the same data source.

##### Multiple interventions groups and multiple interventions per individuals

Studies with multiple intervention groups with different individuals will be included in this review, although only intervention and control groups that meet the eligibility criteria will be used in the data synthesis. To avoid problems with dependence between effect sizes we will apply robust standard errors (Hedges, Tipton, & Johnson, 2010) and use the small sample adjustment to the estimator itself (Tipton, 2015). We will use the results in Tanner‐Smith and Tipton (2014) and Tipton (2015) to evaluate if there are enough studies for this method to consistently estimate the standard errors. See Section 4.3.11 below for more details about the data synthesis.

If there are not enough studies, we will use a synthetic effect size (the average) in order to avoid dependence between effect sizes. This method provides an unbiased estimate of the mean effect size parameter but overestimates the standard error. Random effects models applied when synthetic effect sizes are involved actually perform better in terms of standard errors than do fixed effects models (Hedges, 2007a). However, tests of heterogeneity when synthetic effect sizes are included are rejected less often than nominal.

If pooling is not appropriate (e.g., the multiple interventions and/or control groups include the same individuals), only one intervention group will be coded and compared to the control group to avoid overlapping samples. The choice of which estimate to include will be based on our risk of bias assessment. We will choose the estimate that we judge to have the least risk of bias (primarily, confounding bias and in case of equal scoring the missing outcome data domain will be used).

##### Multiple studies using the same sample of data

In some cases, several studies may have used the same sample of data or some studies may have used only a subset of a sample used in another study. We will review all such studies, but in the meta‐analysis we will only include one estimate of the effect from each sample of data. This will be done to avoid dependencies between the “observations” (i.e., the estimates of the effect) in the meta‐analysis. The choice of which estimate to include will be based on our risk of bias assessment of the studies. We will choose the estimate from the study that we judge to have the least risk of bias (primarily, confounding bias). If two (or more) studies are judges to have the same risk of bias and one of the studies (or more) uses a subset of a sample used in another study (or studies) we will include the study using the full set of participants.

##### Multiple time points

When the results are measured at multiple time points, each outcome at each time point will be analysed in a separate meta‐analysis with other comparable studies taking measurements at a similar time point. As a general guideline, these will be grouped together as follows: (a) postintervention (b) less than a year follow up, (c) 1–2 year follow up, and (d) More than 2 year follow up. However, should the studies provide viable reasons for an adjusted choice of relevant and meaningful duration intervals for the analysis of outcomes, we will adjust the grouping.

#### 3.3.3 Selection of studies

Under the supervision of review authors, two review team assistants will first independently screen titles and abstracts to exclude studies that are clearly irrelevant. Studies considered eligible by at least one assistant or studies were there is insufficient information in the title and abstract to judge eligibility, will be retrieved in full text. The full texts will then be screened independently by two review team assistants under the supervision of the review authors. Any disagreement of eligibility will be resolved by the review authors. Exclusion reasons for studies that otherwise might be expected to be eligible will be documented and presented in an appendix.

The study inclusion criteria will be piloted by the review authors (see Appendix *First and second level screening*). The overall search and screening process will be illustrated in a flow diagram. None of the review authors will be blind to the authors, institutions or the journals responsible for the publication of the articles.

#### 3.3.4 Data extraction and management

Two review authors will independently code and extract data from included studies. A coding sheet will be piloted on several studies and revised as necessary (see Appendix *Data extraction*). Disagreements will be resolved by consulting a third review author with extensive content and methods expertise. Disagreements resolved by a third reviewer will be reported. Data and information will be extracted on: available characteristics of participants, intervention characteristics and control conditions, research design, sample size, risk of bias and potential confounding factors, outcomes, and results. Extracted data will be stored electronically. Analysis will be conducted using RevMan5 and Stata software.

#### 3.3.5 Assessment of risk of bias in included studies

We will assess the risk of bias in randomised studies using Cochranes revised risk of bias tool, ROB 2 (Higgins et al., 2019).

The tool is structured into five domains, each with a set of signalling questions to be answered for a specific outcome. The five domains cover all types of bias that can affect results of randomised trials.

The five domains for individually randomised trials are:

(1) Bias arising from the randomisation process;
(2) Bias due to deviations from intended interventions (separate signalling questions for effect of assignment and adhering to intervention);
(3) Bias due to missing outcome data;
(4) Bias in measurement of the outcome;
(5) Bias in selection of the reported result.

For cluster‐randomised trials, an additional domain is included ((1b) Bias arising from identification or recruitment of individual participants within clusters). We will use the latest template for completion (currently it is the version of March 15, 2019 for individually randomised parallel‐group trials and October 20, 2016 for cluster randomised parallel‐group trials). In the cluster randomised template however, only the risk of bias due to deviation from the intended intervention (effect of assignment to intervention; intention to treat) is present and the signalling question concerning the appropriateness of the analysis used to estimate the effect is missing. Therefore, for cluster randomised trials we will only use the signalling questions concerning the bias arising from identification or recruitment of individual participants within clusters from the template for cluster randomised parallel‐group trials; otherwise we will use the template and signalling questions for individually randomised parallel‐group trials.

We will assess the risk of bias in nonrandomised studies, using the model ROBINS‐I, developed by members of the Cochrane Bias Methods Group and the Cochrane Non‐Randomised Studies Methods Group (Sterne, Hernán, et al., 2016). We will use the latest template for completion (currently it is the version of September 19, 2016).

The ROBINS‐I tool is based on the Cochrane RoB tool for randomised trials, which was launched in 2008 and modified in 2011 (Higgins et al., 2011).

The ROBINS‐I tool covers seven domains (each with a set of signalling questions to be answered for a specific outcome) through which bias might be introduced into nonrandomised studies:

(1) Bias due to confounding
(2) Bias in selection of participants
(3) Bias in classification of interventions
(4) Bias due to deviations from intended interventions;
(5) Bias due to missing outcome data;
(6) Bias in measurement of the outcome;
(7) Bias in selection of the reported result.

The first two domains address issues before the start of the interventions and the third domain addresses classification of the interventions themselves. The last four domains address issues after the start of interventions and there is substantial overlap for these four domains between bias in randomised studies and bias in nonrandomised studies trials (although signalling questions are somewhat different in several places, see Sterne, Higgins, et al., 2016 and Higgins et al., 2019).

Randomised study outcomes are rated on a “Low/Some concerns/High” scale on each domain; whereas nonrandomised study outcomes are rated on a “Low/Moderate/Serious/Critical/No Information” scale on each domain. The level “Critical” means: the study (outcome) is too problematic in this domain to provide any useful evidence on the effects of intervention and it is excluded from the data synthesis. The same critical level of risk of bias (excluding the result from the data synthesis) is not directly present in the RoB 2 tool, according to the guidance to the tool (Higgins et al., 2019).

We will add a critical level of risk of bias to the RoB 2 tool with the same meaning as in the ROBINS‐I tool; that is, the study (outcome) is too problematic in this domain to provide any useful evidence on the effects of intervention and it is excluded from the data synthesis. We will stop the assessment of a randomised study outcome using the RoB 2 as soon as one domain is judged as “Critical”. Likewise, we will stop the assessment of a nonrandomised study outcome as soon as one domain in the ROBINS‐I is judged as “Critical”.

“High” risk of bias in multiple domains in the RoB 2 assessment tool may lead to a decision of an overall judgement of “Critical” risk of bias for that outcome and it will be excluded from the data synthesis. “Serious” risk of bias in multiple domains in the ROBINS‐I assessment tool may lead to a decision of an overall judgement of “Critical” risk of bias for that outcome and it will be excluded from the data synthesis.

##### Confounding

An important part of the risk of bias assessment of nonrandomised studies is consideration of how the studies deal with confounding factors. Systematic baseline differences between groups can compromise comparability between groups. Baseline differences can be observable (e.g., age and gender) and unobservable (to the researcher; e.g., motivation and “ability”). There is no single nonrandomised study design that always solves the selection problem. Different designs represent different approaches to dealing with selection problems under different assumptions, and consequently require different types of data. There can be particularly great variations in how different designs deal with selection on unobservables. The “adequate” method depends on the model generating participation, that is, assumptions about the nature of the process by which participants are selected into a programme.

A major difficulty in estimating causal effects of outreach work is the potential endogeneity of the young individual's life circumstance that leads to the decision of the outreach worker to reach out to that particular young person and if not accounted for it will yield biased estimates.

As there is no universal correct way to construct counterfactuals for nonrandomised designs, we will look for evidence that identification is achieved, and that the authors of the primary studies justify their choice of method in a convincing manner by discussing the assumption(s) leading to identification (the assumption(s) that make it possible to identify the counterfactual). Preferably the authors should make an effort to justify their choice of method and convince the reader that the only difference between a treated individual and a nontreated individual is the treatment. The judgement is reflected in the assessment of the confounder unobservables in the list of confounders considered important at the outset (see Appendix *User guide for unobservables*).

In addition to unobservables, we have identified the following observable confounding factors to be most relevant: age, gender and risk indicators as described in section *Type of participants*. In each study, we will assess whether these factors have been considered, and in addition we will assess other factors likely to be a source of confounding within the individual included studies.

##### Importance of prespecified confounding factors

The motivation for focusing on age, gender and risk indicators is given below.

The prevalence of different types of behavioural and psychological problems, coping skills, cognitive and emotional ability vary throughout a child's development through puberty and into adulthood (Cole et al., 2005), and therefore we consider age to be a potential confounding factor. Furthermore, there are substantial gender differences in behaviour problems, coping and risk of different types of adverse outcomes which is why we also include gender as a potential confounding factor (Card et al., 2008; Hampel & Petermann, 2005; Hart et al., 2007).

Pretreatment group equivalence of risk indicators is indisputable an important confounder as young people in stable life circumstances, typically are not at risk of developing the range of problems we will consider in this review. Therefore, the accuracy of the estimated effects of outreach programmes will depend crucially on how well the risk indicators are controlled for.

##### Effect of primary interest and important cointerventions

We are mainly interested in the effect of starting and adhering to the intended intervention, that is, the treatment on the treated effect. The risk of bias assessments will therefore be in relation to this specific effect.

As the intervention is outreach to young people who are very unlikely to seek out help for themselves, we cannot think of any important differences in additional interventions (“co‐interventions”) between intervention groups that can bias the estimated effect.

##### Assessment

At least two review authors will independently assess the risk of bias for each relevant outcome from the included studies. Any disagreements will be resolved by a third reviewer with content and statistical expertise and will be reported. We will report the risk of bias assessment in risk of bias tables for each included study outcome in the completed review.

#### 3.3.6 Measures of treatment effect

##### Continuous outcomes

For continuous outcomes, effects sizes with 95% confidence intervals will be calculated, where means and standard deviations are available. If means and standard deviations are not available, we will calculate standardised mean differences (SMDs) from *F* ratios, *t* values, *χ* ^2^ values and correlation coefficients, where available, using the methods suggested by Lipsey and Wilson (2001). If not enough information is yielded, the review authors will request this information from the principal investigators. Hedges' *g* will be used for estimating SMDs. Any measures of drug and alcohol use or social and emotional outcomes, are examples of relevant continuous outcomes in this review.

##### Dichotomous outcomes

For dichotomous outcomes, we will calculate odds ratios with 95% confidence intervals. Delinquency, school failure, gang involvement/membership and homelessness, are examples of relevant dichotomous outcomes in this review.

There are statistical approaches available to re‐express dichotomous and continuous data to be pooled together (Sánchez‐Meca et al., 2003). In order to calculate common metric odds ratios will be converted to SMD effect sizes using the Cox transformation. We will only transform dichotomous effect sizes to SMD if appropriate, for example, as may be the case with, for example, the outcomes drug and alcohol use, that can be measured with binary and continuous data.

When effect sizes cannot be pooled, study‐level effects will be reported in as much detail as possible. Software for storing data and statistical analyses will be RevMan 5.0, Excel, R and Stata 10.0.

#### 3.3.7 Unit of analysis issues

Errors in statistical analysis can occur when the unit of allocation differs from the unit of analysis. In cluster randomised trials, participants are randomised to treatment and control groups in clusters, either when data from multiple participants in a setting are included (creating a cluster within the community setting), or when participants are randomised by treatment locality. Nonrandomised studies may also include clustered assignment of treatment. Effect sizes and standard errors from such studies may be biased if the unit‐of‐analysis is the individual and an appropriate cluster adjustment is not used (Higgins & Green, 2011).

If possible, we will adjust effect sizes individually using the methods suggested by Hedges (2007b) and information about the intracluster correlation coefficient (ICC), realised cluster sizes and/or estimates of the within and between variances of clusters. If it is not possible to obtain this information, we will adjust effect sizes using estimates from the literature (we will search for estimates of relevant ICC's), and assume equal cluster sizes. To calculate an average cluster size, we will divide the total sample size in a study by the number of clusters.

#### 3.3.8 Dealing with missing data

Missing data and attrition rates will be assessed in the included studies; see Section 4.3.5. Where studies have missing summary data, such as missing standard deviations, the review authors will request this information from the principal investigators. If no information is yielded, we will derive these where possible from *F* ratios, *t* values, *χ* ^2^ values and correlation coefficients using the methods suggested by Lipsey and Wilson (2001). If missing summary data cannot be derived, the study results will be reported in as much detail as possible.

#### 3.3.9 Assessment of heterogeneity

Heterogeneity among primary outcome studies will be assessed with *χ* ^2^ (Q) test, and the *I* ^2^ and *τ* ^2^ statistics (Higgins et al., 2003). Any interpretation of the *χ* ^2^ test will be made cautiously on account of its low statistical power.

#### 3.3.10 Assessment of reporting biases

Reporting bias refers to both publication bias and selective reporting of outcome data and results. Here, we state how we will assess publication bias.

We will use funnel plots for information about possible publication bias if we find sufficient studies (Higgins & Green, 2011). However, asymmetric funnel plots are not necessarily caused by publication bias (and publication bias does not necessarily cause asymmetry in a funnel plot). If asymmetry is present, we will consider possible reasons for this.

#### 3.3.11 Data synthesis

The proposed project will follow standard procedures for conducting systematic reviews using meta‐analysis techniques. All follow‐up durations reported in the primary studies will be recorded and we will do separate analyses for short‐term and long‐term outcomes.

The overall data synthesis will be conducted where effect sizes are available or can be calculated, and where studies are similar in terms of the outcome measured. Meta‐analysis of outcomes will be conducted on each metric (as outlined in Section 4.1.4) separately.

As different computational methods may produce effect sizes that are not comparable, we will be transparent about all methods used in the primary studies (research design and statistical analysis strategies) and use caution when synthesising effect sizes. Special caution will be taken concerning studies using regression discontinuity (RD) to estimate a local average treatment effect (LATE; Angrist & Pischke, 2009). These will be included, but may be subject to a separate analysis depending on the comparability between the LATE's and the effects from other studies. We will in any case check the sensitivity of our results to the inclusion of RD studies. In addition, we will discuss the limitation in generalisation of results obtained from these types of studies.

When the effect sizes used in the data synthesis are odds ratios, they will be log transformed before being analysed. The reason is that ratio summary statistics all have the common feature that the lowest value that they can take is 0, that the value 1 corresponds with no intervention effect, and the highest value that an odds ratio can ever take is infinity. This number scale is not symmetric. The log transformation makes the scale symmetric: the log of 0 is minus infinity, the log of 1 is zero, and the log of infinity is infinity.

Studies that have been coded with a critical risk of bias will not be included in the data synthesis.

As the intervention deal with diverse populations of participants (from different countries, facing different curriculums, etc.), and we therefore expect heterogeneity among primary study outcomes, all analyses of the overall effect will be inverse variance weighted using random effects statistical models that incorporate both the sampling variance and between study variance components into the study level weights. Random effects weighted mean effect sizes will be calculated using 95% confidence intervals and we will provide a graphical display (Forest plot) of effect sizes. Graphical displays for meta‐analysis performed on ratio scales sometimes use a log scale, as the confidence intervals then appear symmetric. This is however not the case for the software Revman 5 which we plan to use in this review.^1^ The graphical displays using odds ratios and the mean effect size will be reported as a odds ratio. Heterogeneity among primary outcome studies will be assessed with *χ* ^2^ (Q) test, and the *I* ^2^, and *τ* ^2^ statistics (Higgins et al., 2003). Any interpretation of the *χ* ^2^ test will be made cautiously on account of its low statistical power.

For subsequent analyses of moderator variables that may contribute to systematic variations, we will use the mixed‐effects regression model, if there are a sufficient number of studies. This model is appropriate if a predictor explaining some between‐studies variation is available but there is a need to account for the remaining uncertainty (Hedges & Pigott, 2004; Konstantopoulos, 2006).

Several studies may have used the same sample of data. We will review all such studies, but in the meta‐analysis we will only include one estimate of the effect from each sample of data. This will be done to avoid dependencies between the “observations” (i.e., the estimates of the effect) in the meta‐analysis. The choice of which estimate to include will be based on our quality assessment of the studies. We will choose the estimate from the study that we judge to have the least risk of bias, with particular attention paid to confounding bias.

Studies may provide results separated by, for example, age and/or gender. We will include results for all age and gender groups. To take into account the dependence between such multiple effect sizes from the same study, we will apply robust variance estimation (RVE) approach (Hedges et al., 2010). An important feature of this analysis is that the results are valid regardless of the weights used. For efficiency purposes, we will calculate the weights using a method proposed by Hedges et al. (2010). This method assumes a simple random‐effects model in which study average effect sizes vary across studies (*τ* ^2^) and the effect sizes within each study are equicorrelated (*ρ*). The method is approximately efficient, since it uses approximate inverse‐variance weights: they are approximate given that *ρ* is, in fact, unknown and the correlation structure may be more complex. We will calculate weights using estimates of *τ* ^2^, setting *ρ* = 0.80 and conduct sensitivity tests using a variety of *ρ* values; to asses if the general results and estimates of the heterogeneity is robust to the choice of *ρ*. We will use the small sample adjustment to the residuals used in RVE as proposed by Bell and McCaffrey (2002) and extended by McCaffrey et al. (2001) and by Tipton (2015). We will use the Satterthwaite degrees of freedom (Satterthwaite, 1946) for tests as proposed by Bell and McCaffrey (2002) and extended by Tipton (2015). We will use the guidelines provided in Tanner‐Smith and Tipton (2014) to evaluate if there are enough studies for this method to consistently estimate the standard errors.

If there is not a sufficient number of studies to use RVE we will conduct a data synthesis where we use a synthetic effect size (the average) in order to avoid dependence between effect sizes.

#### 3.3.12 Subgroup analysis and investigation of heterogeneity

We will investigate the following factors with the aim of explaining potential observed heterogeneity: study‐level summaries of participant characteristics (e.g., studies considering a specific gender or age group or studies where separate effects for girls/boys or age groups (e.g., 8–17 year old/18–25 year old) are available) and target group (if the programme is targeted towards a specific risky behaviour such as, e.g., homeless youth, youth who are on active probation or youth who are gang‐involved etc.).

If the number of included studies is sufficient and given there is variation in the covariates (age, gender and target group), we will perform moderator analyses (multiple meta‐regression using the mixed model) to explore how observed variables are related to heterogeneity.

If there are a sufficient number of studies, we will apply the RVE approach and use approximately inverse variance weights calculated using a method proposed by Hedges et al. (2010). This technique calculates standard errors using an empirical estimate of the variance: it does not require any assumptions regarding the distribution of the effect size estimates. The assumptions that are required to meet the regularity conditions are minimal and generally met in practice. This more robust technique is beneficial because it takes into account the possible correlation between effect sizes separated by the covariates within the same study (e.g., age or gender separated effects) and allows all of the effect size estimates to be included in meta‐regression. We will calculate weights using estimates of *τ* ^2^, setting *ρ* = 0.80 and conduct sensitivity tests using a variety of *ρ* values; to asses if the general results is robust to the choice of *ρ*. We will use the small sample adjustment to the residuals used in RVE and the Satterthwaite degrees of freedom (Satterthwaite, 1946) for tests (Tipton, 2015). The results in Tipton (2015) suggests that the degrees of freedom depend on not only the number of studies but also on the type of covariates included in the meta‐regression. The degrees of freedom can be small, even when the number of studies is large if a covariate is highly unbalanced or a covariate with very high leverage is included, The degrees of freedom will vary from coefficient to coefficient. The corrections to the degrees of freedom enable us to assess when the RVE method performs well. As suggested by Tanner‐Smith and Tipton (2014) and Tipton (2015) if the degrees of freedom are smaller than four, the RVE results should not be trusted.

We will report 95% confidence intervals for regression parameters. We will estimate the correlations between the covariates and consider the possibility of confounding. Conclusions from meta‐regression analysis will be cautiously drawn and will not solely be based on significance tests. The magnitude of the coefficients and width of the confidence intervals will be taken into account as well. Otherwise, single factor subgroup analysis will be performed. The assessment of any difference between subgroups will be based on 95% confidence intervals. Interpretation of relationships will be cautious, as they are based on subdivision of studies and indirect comparisons.

In general, the strength of inference regarding differences in treatment effects among subgroups is controversial. However, making inferences about different effect sizes among subgroups on the basis of between‐study differences entails a higher risk compared to inferences made on the basis of within study differences; see Oxman and Guyatt (1992). We will therefore use within study differences where possible.

We will also consider the degree of consistence of differences, as making inferences about different effect sizes among subgroups entails a higher risk when the difference is not consistent within the studies; see Oxman and Guyatt (1992).

#### 3.3.13 Sensitivity analysis

Sensitivity analysis will be carried out by restricting the meta‐analysis to a subset of all studies included in the original meta‐analysis and will be used to evaluate whether the pooled effect sizes are robust across components of risk of bias. We will consider sensitivity analysis for each domain of the risk of bias checklists and restrict the analysis to studies with a low risk of bias.

Sensitivity analyses with regard to research design and statistical analysis strategies in the primary studies will be an important element of the analysis to ensure that different methods produce consistent results.

##### Treatment of qualitative research

We do not plan to include qualitative research.

## CONTRIBUTIONS OF AUTHORS

- Content: T. F. and N. T. D.
- Systematic review methods: T. F. and N. T. D.
- Statistical analysis: T. F.
- Information retrieval: B. C. A. V.

## DECLARATIONS OF INTEREST

There are no potential conflicts of interest.

### Preliminary timeframe

Approximate date for submission of the systematic review will be no longer than two years after protocol approval.

### Plans for updating this review

Trine Filges will be responsible for updating the review and updates can be expected each second year.

## SOURCES OF SUPPORT

### Internal sources

- No sources of support provided

### External sources

- VIVE Campbell, Denmark

## Footnotes

1 If we apply robust variance estimation, the analysis will be conducted in STATA or Ras robust variance estimation is not implemented in Revman 5.

## APPENDIX A

### First and second level screening

First level screening is on the basis of titles and abstracts. Second level is on the basis of full text

Reference id. No.:

Reviewers initials:

Source:

Year of publication:

Country/countries of origin:

Author(s):

The study will be excluded if one or more of the answers to question 1–3 are “No”. If the answers to question 1–3 are “Yes” or “Uncertain”, then the full text of the study will be retrieved for second level eligibility. All unanswered questions need to be posed again on the basis of the full text. If not enough information is available, or if the study is unclear, the author of the study will be contacted if possible.

**Screening questions:**

1. Does the study focus on outreach work?
  Yes—include.
  No—if no then stop here and exclude.
  Uncertain—include.
  Question 1 guidance:
  The intervention in this review is outreach work which may also be termed detached youth work, street work or fieldwork. Outreach efforts with services only focusing on nutritional and medical care (e.g., testing for HIV) will be excluded.
2. Are the participants young people between 8 and 25 years of age living in OECD countries, who either have experienced or is at‐risk of experiencing an adverse outcome such as school failure, drug use, participation in delinquent behaviours, runaway and homelessness?
  Yes—include.
  No—if no then stop here and exclude.
  Uncertain—include
  Question 2 guidance:
  At‐risk may be based on such indicators as the young person's level of association with negative peers (e.g., negative attitudes toward school and poor educational outlook, gang members, etc.), hanging out on the streets or in gang neighbourhoods, poor academic history, coming from a highly distressed or crisis ridden, low income family in a racially/ethnically segregated neighbourhood, and prior involvement in illegal and delinquent activities.
3. Is the report/article a quantitative evaluation study with a comparison condition?

Yes—include

No—if no then stop here and exclude

Uncertain—include

Question 3 guidance:

We are only interested in primary quantitative studies with a comparison group, where the authors have analysed the data. We are not interested in theoretical papers on the topic or surveys/reviews of studies of the topic (this question may be difficult to answer on the base of titles and abstracts alone).

### Data extraction

**Table:**

**Names of author(s)**
**Title**
**Language**
**Journal**
**Year**
**Country**
**Target group**
**Participant characteristic (age, gender, ethnicity, risk factors)**
**Services combined with the outreach component** (e.g., case management or participation in community programmes or a continuum of comprehensive services including education, employment and intensive supervision)
**Duration** (number of weeks, months or years)
**Intensity** (number of hours per week/month)
**Type of data used in study (administrative, questionnaire, other (specify))**
**Time period covered by analysis (divide into intervention and follow up)**
**Sample size (divide into treated/comparison)**

#### Outcome measures

Instructions: Please enter outcome measures in the order in which they are described in the report. Note that a single outcome measure can be completed by multiple sources and at multiple points in time (data from specific sources and time‐points will be entered later).

**Table:**

#	Outcome and measure	Reliability and validity	Format	Direction	Pg# & notes
1		Info from:	Dichotomy	High score or event is	
		Other samples	Continuous	Positive	
				Negative	
		This sample		Can't tell	
		Unclear			
		Info provided:			

*Repeat as needed

#### OUT COME DATA

##### DICHOTOMOUS OUTCOME DATA

**Table:**

Outcome	Time point(s) (record exact time from participation, there may be more than one, record them all)	Source	Valid Ns	Cases	Noncases	Statistics	Pg. # & Notes
		Questionnaire	Participation	Participation	Participation	RR (risk ratio)	
		Admin data				OR (odds ratio)	
		Other (specify)	Comparison	Comparison	Comparison	SE (standard error)	
						95% CI	
		Unclear				DF	
						*p* value (enter exact *p* value if available)	
						*χ* ^2^	
						Other	

Repeat as needed

##### CONTINUOUS OUTCOME DATA

**Table:**

Outcome	Time point(s) (record exact time from participation, there may be more than one, record them all)	Source (specify)	Valid Ns	Means	SDs	Statistics	Pg. # & Notes
		Questionnaire	Participation	Participation	Participation	*p*	
		Admin data				*t*	
			Comparison	Comparison	Comparison	*F*	
		Other (specify)				Df	
		Unclear				ES	
						Other	

*Repeat as need

### Assessment of risk of bias in included studies

#### User guide for unobservables

Systematic baseline differences between groups can compromise comparability between groups. Baseline differences can be observable (e.g., age and gender) and unobservable (to the researcher; e.g., motivation and “ability”). There is no single nonrandomised study design that always solves the selection problem. Different designs solve the selection problem under different assumptions and require different types of data. Especially how different designs deal with selection on unobservables varies. The “right” method depends on the model generating participation, that is, assumptions about the nature of the process by which participants are selected into a programme.

As there is no universal correct way to construct counterfactuals we will assess the extent to which the identifying assumptions (the assumption that makes it possible to identify the counterfactual) are explained and discussed (preferably the authors should make an effort to justify their choice of method). We will look for evidence that authors using, for example (this is NOT an exhaustive list):

##### Natural experiments

Discuss whether they face a truly random allocation of participants and that there is no change of behaviour in anticipation of, for example, policy rules.

##### Matching (including propensity scores)

Explain and discuss the assumption that there is no selection on unobservables, only selection on observables.

##### (Multivariate, multiple) regression

Explain and discuss the assumption that there is no selection on unobservables, only selection on observables. Further discuss the extent to which they compare comparable people.

##### Regression discontinuity

Explain and discuss the assumption that there is a (strict!) RD treatment rule. It must not be changeable by the agent in an effort to obtain or avoid treatment. Continuity in the expected impact at the discontinuity is required.

##### Difference‐in‐difference (treatment‐control‐before‐after)

Explain and discuss the assumption that the trends in treatment and control groups would have been parallel, had the treatment not occurred.

#### Justification of exclusion of studies using an instrumental variable (IV) approach

Studies using IVs for causal inference in nonrandomised studies will not be included as the interpretation of IV estimates is challenging. IV only provides an estimate for a specific group namely, people whose behaviour change due to changes in the particular instrument used. It is not informative about effects on never‐takers and always‐takers because the instrument does not affect their treatment status. The estimated effect is thus applicable only to the subpopulation whose treatment status is affected by the instrument. As a consequence, the effects differ for different IVs and care has to be taken as to whether they provide useful information. The effect is interesting when the instrument it is based on is interesting in the sense that it corresponds to a policy instrument of interest. Further, if those that are affected by the instrument are not affected in the same way the IV estimate is an average of the impacts of changing treatment status in both directions, and cannot be interpreted as a treatment effect. To turn the IV estimate into a LATE requires a monotonicity assumption. The movements induced by the instrument go in one direction only, from no treatment to treatment. The IV estimate, interpreted as a LATE, is only applicable to the complier population, those that are affected by the instrument in the “right way”. It is not possible to characterise the complier population as an observation's subpopulation cannot be determined and defiers do not exist by assumption.

In the binary‐treatment–binary‐instrument context, the IV estimate can, given monotonicity, be interpreted as a LATE; that is, the average treatment effect for the subpopulation of compliers. If treatment or instruments are not binary, interpretation becomes more complicated. In the binary‐treatment–multivalued‐instrument (ordered to take values from 0 to *J*) context, the IV estimate, given monotonicity, is a weighted average of pairwise LATE parameters (comparing subgroup *j* with subgroup *j* − 1). The IV estimate can thus be interpreted as the weighted average of average treatment effects in each of the *J* subgroups of compliers. In the multivalued‐treatment (ordered to take values from 0 to *T*) − multivalued‐instrument (ordered to take values from 0 to *J*) context, the IV estimate for *each pair of instrument values*, given monotonicity, is a weighted average of the effects from going from *t* − 1 to *t* for persons induced by the change in the value of the instrument to move from any level below *t* to the level *t* or any level above. Persons can be counted multiple times in forming the weights. (Angrist, & Pischke, 2009; Heckman & Urzúa, 2010; Heckman, et al., 2006).

## References

[cl21121-bib-0002] Altena, A. M. , Brilleslijper‐Kater, S. N. , & Wolf, J. R. L. M. (2010). Effective interventions for homeless youth a systematic review. American Journal of Preventive Medicine, 38(6), 637–45. 20494240 10.1016/j.amepre.2010.02.017

[cl21121-bib-0003] Angrist, J. D. , & Pischke, J. S. (2009). Mostly harmless econometrics: An empiricist's companion. Princeton, NJ: Princeton University Press.

[cl21121-bib-0004] Arbreton, A. J. , Sheldon, J. , & Herrera, C. (2005). *Beyond safe havens: A synthesis of 20 years of research on the boys & girls clubs* (Full Report). Philadelphia, PA: Public/Private Ventures.

[cl21121-bib-0005] Arbreton, A. J. A. , & McClanahan, W. S. (2002). *Targeted outreach: Boys & Girls Clubs of America's approach to gang prevention and intervention*. Public/Private Ventures, Philadelphia, PA.

[cl21121-bib-0006] Bell, R. M. , & McCaffrey, D. F. (2002). Bias reduction in standard errors for linear regression with multi‐stage samples. Survey Methodology, 28(2), 169–181.

[cl21121-bib-0007] Benjaminsen, L. (2019). Hjemløshed i Danmark 2019—National kortlægning. Copenhagen: VIVE—The Danish Center for Social Science Research, Report 09:19 Retrieved from https://www.vive.dk/da/temaer/hjemloeshed/udgivelser/hjemloeshed-i-danmark-2019-14218/

[cl21121-bib-0008] Benjaminsen, L. , Enemark, M. H. , & Jeppesen, T. (2020). 15045/. Hjemløshed i ungdommen—En registerbaseret undersøgelse af unges forløb før og efter en hjemløshedssituation. Copenhagen: VIVE—The Danish Center for Social Science Research, Report 24:20.Retrieved from https://www.vive.dk/da/udgivelser/hjemloeshed-i-ungdommen-15045/

[cl21121-bib-0010] Campie, P. E. , Vriniotis, M. , Read, N. W. , Fronius, T. , & Petrosino, A. (2014). A comparative study using propensity score matching to predict incarceration likelihoods among SSYI and non‐SSYI youth from 2011–2013.

[cl21121-bib-0011] Card, N. A. , Stucky, B. D. , Sawalani, G. M. , & Little, T. D. (2008). Direct and indirect aggression during childhood and adolescence: A meta‐analytic review of gender differences, intercorrelations, and relations to maladjustment. Child Development, 79(5), 1185–229. 18826521 10.1111/j.1467-8624.2008.01184.x

[cl21121-bib-0012] Cohen, M. A. (1998). The monetary value of saving a high‐risk youth. Journal of Quantitative Criminology, 14(1), 5–33.

[cl21121-bib-0013] Cole, M. , Cole, S. R. , & Lightfoot, C. (2005). The development of children. New York: Worth Publishers.

[cl21121-bib-0014] Connolly, J. A. , & Joly, L. E. (2012). Outreach with street‐involved youth: A quantitative and qualitative review of the literature. Clinical Psychology Review, 32, 524–34. 22728669 10.1016/j.cpr.2012.05.006

[cl21121-bib-0015] Coren, E. , Hossain, R. , Pardo Pardo, J. , & Bakker, B. (2016). Interventions for promoting reintegration and reducing harmful behaviour and lifestyles in street‐connected children and young people. Cochrane Database of Systematic Reviews, 1, 1–156. 10.1002/14651858.CD009823.pub3 PMC7096770 26760047

[cl21121-bib-0017] DuBois, D. L. , Holloway, B. E. , Valentine, J. C. , & Cooper, H. (2002). Effectiveness of mentoring programs for youth: A meta‐analytic review. American Journal of Community Psychology, 30(2), 157–97. 12002242 10.1023/A:1014628810714

[cl21121-bib-0018] DuBois, D. L. , Portillo, N. , Rhodes, J. E. , Silverthorn, N. , & Valentine, J. C. (2011). How effective are mentoring programs for youth? A systematic assessment of the evidence. Psychological Science in the Public Interest, 12(2), 57–91. 26167708 10.1177/1529100611414806

[cl21121-bib-0019] Hampel, P. , & Petermann, F. (2005). Age and gender effects on coping in children and adolescents. Journal of Youth and Adolescence, 34(2), 73–83.

[cl21121-bib-0020] Hart, J. L. , O'Toole, S. K. , Price‐Sharps, J. L. , & Shaffer, T. W. (2007). The risk and protective factors of violent juvenile offending: An examination of gender differences. Youth Violence and Juvenile Justice, 5(4), 367–384.

[cl21121-bib-0021] Heckman, J. J. , & Urzúa, S. (2010). Comparing IV with structural models: What simple IV can and cannot identify. Journal of Econometrics, 156, 27–37. 20440375 10.1016/j.jeconom.2009.09.006 PMC2861784

[cl21121-bib-0022] Heckman, J. J. , Urzúa, S. , & Vytlacil, E. (2006). Understanding instrumental variables in models with essential heterogeneity. The Review of Economics and Statistics, 88(3), 389–432.

[cl21121-bib-0023] Hedges, L. V. (2007a). Meta‐analysis. In C. R. Rao (Ed.), The handbook of statistics (pp. 919–953). Amsterdam: Elsevier.

[cl21121-bib-0024] Hedges, L. V. (2007b). Effect sizes in cluster‐randomized designs. Journal of Educational and Behavioral Statistics, 32(4), 341–370.

[cl21121-bib-0025] Hedges, L. V. , Tipton, E. , & Johnson, M. C. (2010). Robust variance estimation in meta‐regression with dependent effect size estimates. Research Synthesis Methods, 1, 39–65. 26056092 10.1002/jrsm.5

[cl21121-bib-0026] Hedges, L. W. , & Pigott, T. D. (2004). The power of statistical tests for moderators in meta‐analysis. Psychological Methods, 9(4), 426–45. 15598097 10.1037/1082-989X.9.4.426

[cl21121-bib-0027] Higgins, J. P. , Thompson, S. G. , Deeks, J. J. , & Altman, D. G. (2003). Measuring inconsistency in meta‐analyses. British Medical Journal, 327(7414), 557–560. 12958120 10.1136/bmj.327.7414.557 PMC192859

[cl21121-bib-0028] Higgins, J. P. T. , Altman, D. G. , Gotzsche, P. C. , Juni, P. , Moher, D. , Oxman, A. D. , … Sterne, J. A. C. (2011). The Cochrane collaboration's tool for assessing risk of bias in randomised trials. BMJ, 343, d5928. 22008217 10.1136/bmj.d5928 PMC3196245

[cl21121-bib-0029] Higgins, J. P. T. & Green, S. (Eds.). (2011). Cochrane handbook for systematic reviews of interventions. Version 5.1.0 [updated March 2011]. New Jersey: Wiley‐Blackwell, The Cochrane Collaboration.

[cl21121-bib-0030] Higgins, J. P. T. , SavoviÄ, J. , Page, M. J. , & Sterne, J. A. C. (editors on behalf of the ROB2 Development Group). Revised Cochrane risk‐of‐bias tool for randomized trials (RoB 2): Detailed guidance. updated 15 March 2019.

[cl21121-bib-0032] Konstantopoulos, S. (2006). *Fixed and mixed effects models in meta‐analysis*.

[cl21121-bib-0033] Korf, D. , Riper, H. , Freeman, M. , Lewis, R. , Grant, I. , Jacob, E. , … Nilson, M. (1999). *Outreach work among drug users in Europe: Concepts, practice and terminology* (Insights Series Number 2).

[cl21121-bib-0034] Lipsey, M. W. , & Wilson, D. B. (2001). Practical meta‐analysis. Thousand Oaks, CA: Sage Publications, Inc.

[cl21121-bib-0036] McCaffrey, D. F. , Bell, R. M. , & Botts, C. H. Generalizations of biased reduced linearization. 2001.

[cl21121-bib-0037] McClanahan, W. S. , Kauh, T. J. , Manning, A. E. , Campos, P. , & Farley, C. (2012). Illuminating solutions: The youth violence reduction partnership. Philadelphia, PA: Public/Private Ventures.

[cl21121-bib-0038] Naranbhai, V. , Abdool Karim, Q. , & Meyer‐Weitz, A. (2011). Interventions to modify sexual risk behaviours for preventing HIV in homeless youth. Cochrane Database of Systematic Reviews, 1, 1–37. 10.1002/14651858.CD007501.pub2 PMC3624078 21249691

[cl21121-bib-0039] NCSL (2019, June 18). *Youth homelessness overview*. Retrieved from https://www.ncsl.org/research/human-services/homeless-and-runaway-youth.aspx

[cl21121-bib-0040] OECD. (2012). Equity and quality in education: Supporting disadvantaged students and schools. OECD Publishing. 10.1787/9789264130852-en

[cl21121-bib-0041] OECD . (2020a). *OECD affordable housing database*. Retrieved from http://oe.cd/ahd. *Indicator HC3.1 homeless population* (last updated on 03/03/2020). Retrieved from https://www.oecd.org/els/family/HC3-1-Homeless-population.pdf

[cl21121-bib-0042] OECD. (2020b). Youth not in employment, education or training (NEET) (indicator). 10.1787/72d1033a-en

[cl21121-bib-0043] Oxman, A. D. , & Guyatt, G. H. (1992). A consumer's guide to subgroup analyses. Annals of Internal Medicine, 116(1), 78–84. 1530753 10.7326/0003-4819-116-1-78

[cl21121-bib-0044] Parker, B. P. (2011). From good intentions to strategic interventions.

[cl21121-bib-0045] Perkins, D. D. , & Zimmerman, M. A. (1995). Empowerment theory, research, and application. American Journal of Community Psychology, 23(5), 569–579. 8851340 10.1007/BF02506982

[cl21121-bib-0046] Quinn, J. (1999). Where need meets opportunity: Youth development programs for early teens. The Future of Children, 9(2), 96–116. 10646262

[cl21121-bib-0047] Rappaport, J. (1985). The power of empowerment language. Social Policy, 15–21.

[cl21121-bib-0048] Ronel, N. (2006). When good overcomes bad: The impact of volunteers on those they help. Human Relations, 59(8), 1133–1153.

[cl21121-bib-0049] Satterthwaite, F. (1946). An approximate distribution of estimates of variance components. Biometrics, 2, 110–114. 20287815

[cl21121-bib-0050] Slesnick, N. , Dashora, P. , Letcher, A. , Erdem, G. , & Serovich, J. (2009). A review of services and interventions for runaway and homeless youth: Moving forward. Children and Youth Services Review, 31, 732–742. 20161294 10.1016/j.childyouth.2009.01.006 PMC2699020

[cl21121-bib-0051] Sterne, J. A. , Hernán, M. A. , Reeves, B. C. , Savović, J. , Berkman, N. D. , Viswanathan, M. , … Higgins, J. P. (2016). ROBINS‐I: A tool for assessing risk of bias in non‐randomized studies of interventions. BMJ, 355, i4919. 27733354 10.1136/bmj.i4919 PMC5062054

[cl21121-bib-0052] Sterne, J. A. C. , Higgins, J. P. T. , Elbers, R. G. , & Reeves, B. C. (2016). *The development group for ROBINS‐I. Risk of bias in non‐randomized studies of interventions (ROBINS‐I): Detailed guidance*. Updated 12 October 2016.

[cl21121-bib-0053] Svensson, N. P. (2003). *Outreach work with young people, young drug users and young people at risk: Emphasis on secondary prevention* [Online]. Strasbourg, France.

[cl21121-bib-0054] Sánchez‐Meca, J. , Marín‐Martínez, F. , & Chacón‐Moscoso, S. (2003). Effect‐size indices for dichotomized outcomes in meta‐analysis. Psychological Methods, 8(4), 448–467. 14664682 10.1037/1082-989X.8.4.448

[cl21121-bib-0055] Tanner‐Smith, E. E. , & Tipton, E. (2014). Robust variance estimation with dependent effect sizes: Practical considerations including a software tutorial in Stata and SPSS. Research Synthesis Methods, 5(1), 13–30. 26054023 10.1002/jrsm.1091

[cl21121-bib-0057] Tipton, E. (2015). Small sample adjustments for robust variance estimation with meta‐regression. Psychological Methods, 20(3), 375–393. 24773356 10.1037/met0000011

[cl21121-bib-0058] Treskon, L. (2016). *What works for disconnected young people: A scan of the evidence* (Working Paper).

[cl21121-bib-0059] Tolan, P. , Henry, D. , Schoeny, M. , & Bass, A. (2008). Mentoring interventions to affect juvenile delinquency and associated problems. Campbell Systematic Reviews, 16.

[cl21121-bib-0060] Xiang, X. (2013). A review of interventions for substance use among homeless youth. Research on Social Work Practice, 23(1), 34–45.

[cl21121-bib-0061] Zimmerman, M. A. (2002). Empowerment theory. In J. Rappaport & E. Seidman (Eds.), Handbook of community psychology (pp. 43–63). Boston, MA: Springer.

[cl21121-bib-0062] Zimmerman, M. A. , Stoddard, S. A. , Eisman, A. B. , Caldwell, C. H. , Aiyer, S. M. , & Miller, A. (2013). Adolescent resilience: Promotive factors that inform prevention. Child Development Perspectives, 7(4), 215–220. 10.1111/cdep.12042 PMC3839856 24288578

